# Associations Between Physical Activity, Body Dissatisfaction, and Mindful Eating in Turkish Adults

**DOI:** 10.3390/nu18081292

**Published:** 2026-04-20

**Authors:** Dilay Ermiş, Rana Turgut Yaman, Ece Akgül, Asu Şevval İçelli, Ceren İşeri, Elif Öztürk, Merve Kızıl, Sude Melis Şahin, Beyzanur Çamlıbel, Gamze Akbulut

**Affiliations:** Department of Nutrition and Dietetics, Faculty of Health Sciences, Istanbul Kent University, Istanbul 34406, Turkey

**Keywords:** physical activity, body dissatisfaction, mindful eating, psychosocial factors

## Abstract

Objective: This cross-sectional study examined the associations among physical activity, body dissatisfaction, and mindful eating in adults, while accounting for the influence of demographic and socioeconomic factors. Methods: A total of 9838 adults (60.6% women, 39.4% men; mean age 36.3 ± 16.0 years) were included in the study using a convenience sampling method (women: 34.4 ± 15.1 years; men: 39.2 ± 16.9 years). Physical activity was measured with the International Physical Activity Questionnaire-Short Form (IPAQ-SF), body dissatisfaction was measured with the Stunkard Figure Rating Scale (FRS), and mindful eating was assessed with the Four Facet Mindful Eating Scale (FFaMES). Spearman correlation, Kruskal–Wallis, and hierarchical regression analyses were conducted. Results: Physical activity showed weak but statistically significant positive correlations with body dissatisfaction and mindful eating, while no association was observed between body dissatisfaction and mindful eating. After adjustment for demographic and socioeconomic variables, body dissatisfaction and mindful eating remained associated with physical activity, although the explained variance was small. Participants with moderate physical activity levels had higher mindful eating scores than those with low activity. Conclusions: The findings indicate weak associations among physical activity, body dissatisfaction, and mindful eating among adults. Although statistically significant, these associations were small, suggesting that these psychosocial factors represent only a limited component of the broader determinants of physical activity behaviour.

## 1. Introduction

Physical activity (PA) is widely recognised as a cornerstone of public health. It plays a critical role in reducing the risk of chronic diseases, including cardiovascular disease, type 2 diabetes, and certain cancers. The World Health Organisation recommends that adults engage in at least 150–300 min of moderate-intensity aerobic physical activity per week to achieve substantial health [1,2]. In addition to its physiological benefits, regular physical activity has been associated with improvements in psychological well-being, including enhanced mood, self-esteem, and overall quality of life [2]. Participation in physical activity has also been shown to positively influence individuals’ perceptions of their bodies and their overall self-concept [3,4].

However, participation in PA is influenced by several psychological and behavioural factors. One such factor is body image, a multidimensional construct encompassing individuals’ perceptions, thoughts, and feelings about their body’s appearance and function [4]. The contemporary literature distinguishes between negative body image, commonly referred to as body dissatisfaction, and positive body image, which reflects body appreciation and acceptance despite perceived imperfections [5]. Previous research has demonstrated a complex and reciprocal relationship between body image and physical activity. While body dissatisfaction may discourage individuals from engaging in physical activity due to body-related concerns or embarrassment, participation in physical activity may also contribute to improvements in body image and self-perception [4,6]. Furthermore, body dissatisfaction has been associated with unhealthy eating behaviours and weight-control practices, as individuals may attempt to modify their appearance to conform to internalised body ideals [5,7]. Evidence also suggests that body image, eating behaviour, and physical activity are interconnected constructs that influence one another within broader health-behaviour frameworks [3,4].

Another psychological construct increasingly recognised as relevant to health-related behaviours is mindfulness. Mindfulness refers to a state of awareness characterised by attention to present-moment experiences with an attitude of openness and non-judgement [8,9]. When applied to eating behaviours, this concept is referred to as mindful eating (ME), which involves awareness of internal hunger and satiety cues, recognition of internal signals related to eating, and non-judgmental attention to the eating experience [9,10]. Research indicates that mindful eating is associated with healthier eating patterns and improved self-regulation of food intake [11].

The emerging literature suggests that mindfulness-related processes may also influence broader health behaviours, including dietary patterns and physical activity engagement [9]. Mindfulness-based approaches are thought to improve emotion regulation and increase awareness of internal experiences, which may support healthier behavioural choices and adaptive self-regulation [8,9]. In addition, mindfulness-based interventions have been shown to positively influence eating behaviour and body image through mechanisms such as enhanced emotional regulation and increased body awareness [12,13]. Psychological mechanisms, such as emotion regulation, may therefore play an important role in understanding the relationships among body image, eating behaviours, and health-related lifestyle behaviours [8,14].

Studying these relationships within different cultural contexts is important, as sociocultural body ideals and gender norms may shape individuals’ perceptions of body image and health behaviours [5,12]. In many societies, thinness is promoted as the ideal body type for women. In contrast, muscularity is emphasised for men, which may contribute to body dissatisfaction and influence both eating behaviours and physical activity engagement [4,5,7].

Examining these associations in Turkish adults provides an important cultural context. Sociocultural standards related to body appearance may exert gender-specific pressures that influence body dissatisfaction, eating behaviours, and physical activity engagement. In particular, cultural body ideals emphasising thinness for women and muscularity for men may contribute to body image concerns and related health behaviours. Investigating these relationships in Turkish adults may therefore provide valuable insights into how psychological and behavioural factors interact within different sociocultural contexts.

Although previous studies have examined the associations between physical activity, body dissatisfaction, and mindful eating separately, research integrating these constructs within a single analytical framework remains limited. In particular, few studies have simultaneously investigated how physical activity and body image relate to mindful eating and its specific subdimensions. Moreover, evidence from non-Western adult populations is scarce, which limits the generalisability of existing findings. Addressing these gaps, the present study examines the associations among physical activity, body dissatisfaction, and mindful eating in a large sample of Turkish adults, with particular attention to mindful eating subcomponents. Therefore, the present study aimed to investigate the relationships between physical activity, body dissatisfaction, and mindful eating in Turkish adults.

Based on the existing literature, the following hypotheses were proposed:

**H1:**  *Physical activity is associated with body dissatisfaction.*

**H2:**  *Physical activity is associated with mindful eating.*

**H3:**  *Body dissatisfaction is associated with mindful eating.*

**H4:**  *Body dissatisfaction and mindful eating are associated with physical activity after controlling for demographic and socioeconomic variables.*

## 2. Materials and Methods

### 2.1. Participants and Procedures

Participants were recruited between July 2024 and May 2025 using a convenience sampling strategy, which was further expanded through snowball sampling. Initially, eligible adults were approached through the researchers’ professional and social networks via email, telephone, and social media. These initial participants were then asked to refer other adults in their networks who met the inclusion criteria. Thus, contact information was obtained through researcher networks and participant referrals rather than from commercial or institutional databases.

Data were collected through face-to-face interviews conducted by the research team using a standardised data collection form. Interviews were conducted in appropriate in-person settings (e.g., participants’ homes, workplaces, community settings, or other mutually convenient locations) and each lasted approximately 15–20 min.

The inclusion criteria for participation were being 18 years of age or older, being able to understand and respond to the questionnaires in Turkish, and providing voluntary consent to participate in the study. Individuals who provided incomplete responses to the questionnaires were excluded from the final analyses.

Prior to participation, all individuals were informed of the study’s purpose and procedures. Participants provided informed consent and were assured that their participation was voluntary and that they could withdraw from the study at any time without any negative consequences.

Ethical approval for this research was obtained from the Health Sciences Research and Publication Ethics Committee of Istanbul Kent University (Approval No: 33983) on 2 July 2024, prior to the initiation of data collection. The study was conducted in accordance with the ethical principles of the Declaration of Helsinki.

A post hoc power analysis was conducted using G*Power (version 3.1.9.7) for multiple regression analysis. Assuming a small effect size (f^2^ = 0.02), an alpha level of 0.05, and nine predictors, the available sample size (*n* = 9838) yielded a statistical power of 1.00. Because a convenience sampling strategy was used, the sample may not fully represent the general population; therefore, the generalizability of the findings should be interpreted with caution.

### 2.2. Measures

#### 2.2.1. International Physical Activity Questionnaire—Short Form (IPAQ-SF)

The International Physical Activity Questionnaire (IPAQ) is a standardised tool developed by researchers from various countries with the support of the World Health Organization and the Centers for Disease Control and Prevention to measure physical activity. Two versions of the IPAQ are available: the 31-item long form (IPAQ-LF) and the 9-item short form (IPAQ-SF). The short form records activity at four intensity levels, including vigorous-intensity activities such as aerobics, moderate-intensity activities such as leisure-time cycling, walking, and sitting. To assess test–retest reliability, Pearson’s correlation coefficient was used. Accordingly, the correlation coefficient for total physical activity was found to be r = 0.69. For walking, the coefficient was r = 0.67, for vigorous activity, r = 0.64, and for moderate activity, r = 0.50. The correlation coefficient for sitting time was higher than the others, with r = 0.78 [15]. In the scoring of IPAQ items, 8 Metabolic Equivalent of Task (METs)-min/week are assigned for vigorous activity, 4 METs for moderate activity, and 3.3 METs for walking. The sitting-time item is considered an indicator of sedentary behaviour but is not included in the calculation of the total physical activity score [16]. When evaluating activities, the criterion is that each activity must be performed for at least 10 min at a time. For each activity level, the MET value is multiplied by the number of days and minutes to obtain the “MET-minutes/week” score. The Turkish validity and reliability study of the IPAQ was conducted by Sağlam et al. in 2010 [17].

##### Stunkard Figure Rating Scale (FRS)

One of the most widely used instruments for assessing body dissatisfaction is the Stunkard Figure Rating Scale [18]. The scale presents a series of schematic silhouettes ranging from very thin to very obese. Participants are instructed to select two figures: the one that best represents their current body size (CBS) and the one that reflects their ideal body size (IBS). While CBS is regarded as an indicator of body perception, IBS and the discrepancy between CBS and IBS are taken as measures of body dissatisfaction [19]. Body dissatisfaction was calculated as the discrepancy score (CBS–IBS), ranging from −8 to +8, where positive values indicate a desire to be thinner, negative values indicate a desire to be heavier, and zero indicates satisfaction [20].

#### 2.2.2. The Four Facet Mindful Eating Scale (FFaMES)

The Four Facet Mindful Eating Scale (FFaMES) was originally developed and validated by Carrière et al. (2022) as a multidimensional measure of food-specific awareness, comprising the domains of non-reactance, non-judgement, internal awareness, and external awareness [21]. The Turkish version of the scale was later adapted by Hamurcu et al. [22] demonstrated validity and reliability for assessing mindful eating in young adults. The instrument consists of 29 items rated on a 5-point Likert scale and evaluates mindful eating across four subdimensions: non-reactance, non-judgement, internal awareness, and external awareness. Non-reactance refers to the ability to avoid impulsive responses to eating-related experiences; non-judgement reflects refraining from negative self-evaluation regarding eating; internal awareness assesses awareness of emotional and cognitive processes influencing eating behaviour; and external awareness refers to noticing external cues that affect eating. In the Turkish form, the non-reactance and non-judgement items are reverse-scored. Total scores range from 29 to 145, with higher scores indicating greater mindful eating. In the Turkish adaptation study, the scale demonstrated strong psychometric properties. Exploratory factor analysis supported the original four-factor structure, explaining 57.3% of the total variance, and confirmatory factor analysis indicated good model fit (RMSEA = 0.013, CFI = 0.981, GFI = 0.966, NFI = 0.948, AGFI = 0.961, SRMR = 0.081, χ^2^/df = 1.021). The overall internal consistency of the Turkish version was high (Cronbach’s α = 0.933), with subscale alpha coefficients of 0.912 for non-reactance, 0.820 for non-judgement, 0.816 for internal awareness, and 0.744 for external awareness [22]. Accordingly, in the present study, the Turkish Four-Facet Mindful Eating Scale was used to assess participants’ mindful eating levels, and both total and subscale scores were calculated for analysis. In this study, mindful eating refers to the construct, while FFaMES total and subscale scores represent its measurement.

### 2.3. Statistical Analysis

Descriptive statistics (mean, standard deviation, frequency, percentage) were calculated for all study variables. Normality of continuous variables was assessed using the Kolmogorov–Smirnov test, skewness and kurtosis values, and histogram inspection. Given the large sample size, the Kolmogorov–Smirnov test was significant for most variables. The literature suggests that variables with skewness and kurtosis values within the interval of −1.5 to +1.5 [23], or more liberally within −2.0 to +2.0 [22], can be regarded as approximately normally distributed. Skewness and kurtosis values for body dissatisfaction and mindful eating scores were within acceptable thresholds, indicating approximate normality, whereas IPAQ MET scores exhibited extreme positive skewness. Therefore, non-parametric methods were applied where appropriate. Spearman’s rho correlation coefficients were used to examine associations between physical activity (IPAQ MET), body dissatisfaction, and mindful eating scores. Participants were also categorised into three groups according to IPAQ guidelines (<600 MET-min/week = low, 600–3000 MET-min/week = moderate, >3000 MET-min/week = high). Group differences in body dissatisfaction and mindful eating scores across IPAQ categories were analysed using the Kruskal–Wallis test. To assess significant results, post hoc pairwise comparisons with Bonferroni correction were conducted. To further examine the predictors of physical activity, hierarchical multiple regression analyses were performed. In the first step, demographic and socioeconomic control variables (age, gender, body mass index (BMI), education, and income status) were entered into the model to control for their potential confounding effects when examining the associations between physical activity and body dissatisfaction. In the second step, psychosocial variables, including body dissatisfaction (Stunkard scores) and mindful eating (FFaMES total scores), were added to assess their additional contribution to the variance in physical activity (IPAQ MET). Prior to conducting hierarchical regression analyses, key assumptions were checked. Linearity and homoscedasticity were examined using residual plots, normality of residuals was assessed with histograms, and multicollinearity among predictors was evaluated using Variance Inflation Factors (VIFs), all of which were within acceptable limits (VIF < 2). In addition, to explore potential gender differences in the observed associations, supplementary analyses were conducted separately for women and men. Correlation and hierarchical regression analyses were repeated after stratifying the sample by gender. Statistical significance was set at *p* < 0.05. All analyses were conducted using IBM SPSS Statistics for Windows, Version 30.0 (IBM Corp., Armonk, NY, USA).

## 3. Results

Demographic characteristics of the participants are presented in Table 1. The mean age of the total sample was 36.3 ± 16.0 years, with women being slightly younger (34.4 ± 15.1 years) than men (39.2 ± 16.9 years). The mean BMI was 24.8 ± 4.6 kg/m^2^, and 51.1% of participants had normal weight. More than half of the participants had an undergraduate degree (53.7%), and most reported that their income was equal to their expenditure (53.5%).

Participants’ IPAQ-SF, Stunkard, and FFaMES scores are presented in Table 2. The mean physical activity score measured with the IPAQ-SF was 1401.3 MET-min/week (SD = 1817.6). According to the IPAQ classification, 39.7% of participants were in the low activity group (<600 MET-min/week), 48.5% in the moderate activity group (600–3000 MET-min/week), and 11.8% in the high activity group (>3000 MET-min/week). The mean Stunkard score was −0.49 ± 1.51. The mean total FFaMES score was 94.8 ± 10.3. The subscale mean scores were 34.2 ± 8.5 for non-reactivity scores, 28.8 ± 7.1 for non-judging scores, 14.3 ± 5.1 for awareness of internal cues scores, and 16.7 ± 5.2 for awareness of external cues scores.

Correlation coefficients between IPAQ, Stunkard, and FFaMES scores are shown in Table 3. A weak positive correlation was found between IPAQ and Stunkard scores (ρ = 0.065, *p* < 0.001) as well as between IPAQ and FFaMES total scores (ρ = 0.066, *p* < 0.001). No significant correlation was observed between Stunkard and FFaMES total scores (ρ = −0.013, *p* = 0.184). Strong positive correlations were found between FFaMES total scores and its subscales, such as non-reactivity scores (ρ = 0.679, *p* < 0.001) and non-judging scores (ρ = 0.663, *p* < 0.001). Moderate to strong negative correlations were observed between FFaMES awareness of internal cues scores and both non-reactivity scores (ρ = −0.619, *p* < 0.001) and non-judging scores (ρ = −0.592, *p* < 0.001). In addition, awareness of external cues scores showed a moderate positive correlation with awareness of internal cues scores (ρ = 0.505, *p* < 0.001).

Additional analyses stratified by gender showed a similar pattern of associations in both women and men (see Supplementary Tables S1 and S2). In women, physical activity was weakly positively correlated with body dissatisfaction (ρ = 0.065, *p* < 0.001) and FFaMES total scores (mindful eating) (ρ = 0.042, *p* = 0.001), while no significant association was observed between body dissatisfaction and FFaMES total scores. In men, physical activity was also weakly positively associated with body dissatisfaction (ρ = 0.065, *p* < 0.001) and FFaMES total scores (ρ = 0.066, *p* < 0.001). A small negative association was observed between body dissatisfaction and FFaMES total scores (ρ = −0.037, *p* = 0.020).

Table 4 presents the results of the Kruskal–Wallis tests across IPAQ physical activity categories. The results indicated significant group differences across IPAQ categories for Stunkard score (χ^2^(2) = 46.64, *p* < 0.001), FFaMES total scores (χ^2^(2) = 43.46, *p* < 0.001), FFaMES non-reactivity score (χ^2^(2) = 14.37, *p* < 0.001), and FFaMES awareness of external cues score (χ^2^(2) = 11.01, *p* = 0.004). Post hoc pairwise comparisons with Bonferroni adjustment revealed that Stunkard scores differed significantly between all IPAQ groups (Low–Moderate, *p* = 0.005; Low–High, *p* < 0.001; Moderate–High, *p* < 0.001). Body dissatisfaction was highest in the low physical activity group (Mean = −0.58 ± 1.62), followed by the moderate activity group (Mean = −0.49 ± 1.44), and lowest in the high activity group (Mean = −0.27 ± 1.38). FFaMES total scores were significantly higher in the moderate activity group (Mean = 95.27 ± 10.38) compared to the low activity group (Mean = 94.30 ± 10.17; *p* < 0.001), while the high activity group showed similar levels (Mean = 94.91 ± 10.93). FFaMES non-reactivity scores were also higher in the moderate activity group (Mean = 34.53 ± 8.36) than in the low activity group (Mean = 34.00 ± 8.63; *p* < 0.001), with comparable scores in the high activity group (Mean = 34.02 ± 8.73).

For FFaMES awareness of external cues scores, significant differences were observed between low and moderate activity groups (*p* = 0.011), with higher scores in the moderate activity group (Mean = 17.07 ± 5.10) compared to the low activity group (Mean = 16.45 ± 5.31), while the high activity group showed similar values (Mean = 16.64 ± 5.61). No significant differences were found for FFaMES non-judging scores (χ^2^(2) = 1.47, *p* = 0.480) or FFaMES awareness of internal cues scores (χ^2^(2) = 0.31, *p* = 0.857).

Hierarchical regression analysis was performed to investigate the effects of demographic, socioeconomic, and psychosocial variables on physical activity levels (IPAQ MET) as presented in Table 5. In Model 1, control variables (age, gender, BMI, education, and income status) were entered. The model was significant, R^2^ = 0.033, F (9, 9776) = 37.48, *p* < 0.001, explaining 3.3% of the variance in physical activity. Within these predictors, gender (β = −0.10, t = −9.91, *p* < 0.001, 95% CI [−0.12, −0.08]) emerged as a significant predictor, indicating lower physical activity levels in women compared to men. Income status was also significant (β = −0.02, t = −1.97, *p* = 0.049, 95% CI [−0.04, 0.00]), suggesting that participants with relatively lower income levels reported lower physical activity. Other control variables (age, BMI, and education) were not significantly associated with physical activity.

In Model 2, psychosocial factors were added to the model, including body dissatisfaction (Stunkard scores) and mindful eating (FFaMES Total Score). The model showed a small but statistically significant increase in explained variance, ΔR^2^ = 0.002, *p* = 0.005, with the overall model remaining significant, R^2^ = 0.035, F (15, 9770) = 23.76, *p* < 0.001. Among the psychosocial predictors, body dissatisfaction (β = 0.02, t = 2.30, *p* = 0.021, 95% CI [0.003, 0.037]) and mindful eating total scores (β = 0.06, t = 2.93, *p* = 0.003, 95% CI [0.02, 0.10]) were significant predictors of physical activity. All predictors met the assumptions of hierarchical regression.

Additional hierarchical regression analyses stratified by gender are presented in Supplementary Tables S3 and S4. None of the psychosocial variables significantly predicted physical activity levels after controlling for BMI, education, and income status among women. In contrast, among men, mindful eating emerged as a significant positive predictor of physical activity (β = 0.046, *p* = 0.003), while body dissatisfaction was not statistically significant. These findings suggest that the association between mindful eating and physical activity may be more pronounced among men.

## 4. Discussion

This study examined the associations between physical activity, body dissatisfaction, and mindful eating in a large sample of adults. Overall, the findings indicated weak but statistically detectable associations between physical activity and both body dissatisfaction and mindful eating, whereas no significant association was observed between body dissatisfaction and mindful eating. Thus, the results were partially consistent with the study hypotheses, although the observed effect sizes were small and should be interpreted with caution.

Beyond these findings, the present study contributes to the existing literature in several important ways. While previous research has often examined physical activity, body image, and mindful eating in isolation or in relatively small, predominantly Western samples, the present study simultaneously investigated these constructs and their subcomponents in a large community-based sample of adults from a non-Western population. By adopting a multidimensional analytical approach, the findings extend prior research by demonstrating how physical activity, body dissatisfaction, and distinct facets of mindful eating may be interrelated, albeit modestly, within a broader behavioural context. This integrative perspective provides a more comprehensive understanding of lifestyle-related behaviours and highlights the importance of considering multiple psychosocial factors together rather than in isolation.

The correlation analyses revealed that physical activity (IPAQ scores) showed only weak but significant associations with both body dissatisfaction and mindful eating, suggesting that higher physical activity is associated with slightly lower body dissatisfaction and greater eating awareness. Physical activity has been proposed as a potential intervention to enhance body image. A systematic review concluded that participants’ perceptions of physical competence and body image were positively associated with their engagement in physical activity [23]. Another study found that adolescents with more positive body image were more likely to engage in physical activity [24]. Experimental studies have also shown that individuals who participate in physical activity tend to report a healthier body image (e.g., greater satisfaction or reduced dissatisfaction) than those who are inactive [6]. These associations may be explained by several mechanisms, including improvements in perceived physical competence, enhanced awareness of body functionality, and psychological benefits such as increased self-esteem and well-being, which may contribute to more favourable body-related perceptions. Previous research has also demonstrated a link between mindful eating and physical activity. For example, a study conducted with university staff found that higher mindful eating scores were positively associated with greater physical activity [25]. The present findings indicate that body dissatisfaction and mindful eating may have a statistically detectable relationship with physical activity engagement; however, the magnitude of this relationship appears limited. Given the very small increase in explained variance, caution is warranted in interpreting these variables as meaningful predictors of physical activity. Statistical significance in this context should not be taken as evidence of substantial clinical or practical importance. Rather, the results suggest that these constructs may represent only a minor part of a more complex behavioural framework.

Notably, the present findings revealed no significant correlation between body dissatisfaction and mindful eating in this sample. This suggests that perceptions related to body image and awareness during eating may operate as distinct psychological processes rather than directly related constructs. Although several studies have reported positive associations between mindfulness-based practices and body image outcomes, the existing evidence is mixed. For example, a recent systematic review concluded that although some studies observed improvements in body image following mindfulness-based interventions, others did not report statistically significant effects [12]. These inconsistencies suggest that the relationship between mindful eating and body image may be more complex than previously assumed and may depend on contextual or methodological factors.

In contrast, strong correlations were observed between FFaMES total scores and its subscales, particularly non-reactivity and non-judging scores, indicating a coherent internal structure of the FFaMES, consistent with previous validation studies in both the original [21] and adapted Turkish version [22]. Negative associations were found between awareness of internal cues scores and the non-reactivity and non-judging scores, suggesting that individuals who are more focused on internal eating signals may be less likely to adopt a non-reactive or non-judgmental stance toward their eating experiences. In contrast, a positive correlation was observed between awareness of external and internal cues scores, indicating that individuals who are more attentive to external food-related signals also tend to be more sensitive to their internal hunger and satiety cues. Consistent with our findings that not all mindful eating dimensions were positively related, Bennett and Latner (2022) also reported that only the disinhibition subscale of the FFaMES was inversely associated with loss of control over eating, whereas awareness and external cues showed no significant associations [11]. These results support the view that mindful eating facets may operate differently and not always in the same direction.

Furthermore, participants in the moderate physical activity group obtained significantly higher FFaMES total scores, as well as higher non-reactivity scores and awareness of external cues scores, compared to those in the low activity group, suggesting that moderate physical activity engagement may foster greater mindful eating. This is consistent with prior evidence suggesting that physical activity supports not only body image but also healthier eating awareness [22]. However, no significant group differences were observed for the non-judging and awareness of internal cues subscales, indicating that not all facets of mindful eating are equally influenced by physical activity, which is consistent with Bennett and Latner’s (2022) findings that only certain FFaMES subscales were significantly related to eating outcomes [11].

Hierarchical regression analyses further demonstrated that, beyond demographic and socioeconomic factors, psychosocial variables made a small but significant contribution to the prediction of physical activity levels. Specifically, body dissatisfaction and mindful eating emerged as significant positive predictors of physical activity, even after controlling for age, gender, BMI, education, and income status. These findings suggest that psychological factors related to body perceptions and eating awareness may also influence engagement in physical activity. This pattern has been consistently documented in large-scale epidemiological studies, which highlight socioeconomic and gender disparities in physical activity participation [10]. For example, previous research has suggested that interventions aiming to promote mindful eating should take into account individual psychosocial characteristics, rather than treating participants as a homogeneous group with similar baseline beliefs, abilities, support, and motivation [26]. Similarly, mindful eating was positively related to physical activity, suggesting that awareness-based approaches to eating can extend to broader health-promoting behaviours [27].

Additional analyses stratified by gender showed broadly similar correlation patterns in both women and men. In both groups, physical activity was weakly associated with body dissatisfaction and mindful eating. However, the regression analyses indicated that mindful eating emerged as a significant predictor of physical activity only among men, whereas no psychosocial predictors were significant among women after adjusting for BMI, education, and income status. These findings may suggest that the relationship between eating awareness and physical activity engagement is somewhat more pronounced among men in this sample. Nevertheless, given the small effect sizes observed, these gender differences should be interpreted cautiously and warrant further investigation in future studies. Previous research has reported no significant gender differences in mindful eating [28].

Although the observed associations were statistically significant, the effect sizes were small, suggesting that physical activity, body dissatisfaction, and mindful eating are likely to represent only one part of a more complex network of determinants of health-related behaviours. Therefore, the findings should be interpreted with caution, particularly regarding their direct clinical or practical implications. From a practical perspective, these findings suggest that interventions promoting physical activity may also support healthier body-related perceptions and greater eating awareness. Public health strategies that integrate physical activity promotion with body image and eating awareness approaches may therefore contribute to more holistic lifestyle interventions.

### 4.1. Limitations of the Study

Several limitations of this study should be acknowledged. The cross-sectional design precludes causal inferences and limits the examination of potential bidirectional or mediating relationships among physical activity, body dissatisfaction, and mindful eating. All variables were assessed using self-report measures, which may be subject to recall bias and social desirability effects. Although the sample was large and heterogeneous, participants were recruited from a single non-Western country using a non-probability sampling approach, which may limit the sample’s representativeness and the generalizability of the findings to other cultural contexts. In addition, while several associations reached statistical significance, the observed effect sizes were small, suggesting that the practical or clinical relevance of these relationships at the individual level may be limited. Finally, the use of the short form of the International Physical Activity Questionnaire, although practical for large-scale studies, may yield less precise estimates of physical activity than objective measures such as accelerometers.

### 4.2. Strengths of the Study

This study has several notable strengths. It was conducted in a large, community-based sample of nearly 10,000 adults from a non-Western context, thereby enhancing the robustness of the findings and addressing the relative underrepresentation of non-Western populations in the existing literature. The large sample size provided sufficient statistical power to detect small but consistent associations among physical activity, body dissatisfaction, and mindful eating. In addition, the use of multiple standardised and validated instruments strengthened the reliability of the measurements. By examining physical activity, body dissatisfaction, and mindful eating simultaneously within a single analytical framework, this study extends previous research that has largely investigated these constructs in isolation and offers an integrated perspective with potential relevance for public health and preventive interventions.

## 5. Conclusions

This study highlights the interrelationships between physical activity, body dissatisfaction, and mindful eating in adults. Physical activity was weakly and positively associated with both body dissatisfaction and mindful eating. In addition, gender and income status were associated with physical activity levels. Although these findings suggest that health-related behaviours may be shaped by psychological and socioeconomic factors, the observed effect sizes were small and should be interpreted with caution. Overall, the results should be viewed as indicative of broader behavioural patterns rather than direct causal or clinically meaningful effects. Importantly, this study contributes to the literature by simultaneously examining physical activity, body dissatisfaction, and mindful eating, along with their subcomponents in a large non-Western adult population. These findings suggest the potential value of holistic strategies that integrate body image, eating awareness, and physical activity promotion to foster sustainable lifestyle changes and enhance overall well-being.

## Figures and Tables

**Table 1:** Demographic and Socioeconomic Characteristics of the Study Participants (*n* = 9838) by Gender.

	Women *n* = 5690 (60.6%)		Men *n* = 3878 (39.4%)		Total *n* = 9838 (100%)	
	Frequency (*n*)	Percentage (%)	Frequency (*n*)	Percentage (%)	Frequency (*n*)	Percentage (%)
Age (Avg ± SD)	34.4 ± 15.1		39.2 ± 16.9		36.3 ± 16.0	
BMI (Avg ± SD)	24.1 ± 4.8		25.9 ± 4.0		24.8 ± 4.6	
<18.5 kg/m^2^	434	7.3	58	1.5	492	5.0
18.5–24.9 kg/m^2^	3351	56.2	1677	43.2	5028	51.1
25.0–29.9 kg/m^2^	1435	24.1	1651	42.6	3086	31.4
>30.0 kg/m^2^	740	12.4	492	12.7	1232	12.5
Education Level						
Illiterate	135	2.3	73	1.9	208	2.1
Primary School	544	9.1	374	9.6	918	9.3
Middle School	334	5.6	343	8.8	677	6.9
High School	1233	20.7	1039	26.8	2272	23.1
Undergraduate	3448	57.9	1833	47.3	5281	53.7
Master’s Degree	241	4.0	192	5.0	433	4.4
Doctorate	25	0.4	24	0.6	49	0.5
Income State						
The income is less than his/her expenses	1303	21.9	579	14.9	1882	19.1
The income is equal to his/her expenditure	3264	54.8	2000	51.6	5264	53.5
The income is more than his/her expenses	1393	23.4	1299	33.5	2692	27.4

Avg: average; SD: standard deviation; BMI: body mass index.

**Table 2:** Descriptive Statistics of Physical Activity (IPAQ MET), Body Dissatisfaction (FRS), and Mindful Eating (FFaMES) Scores.

	*n*	Avg.	SD	Min.	Max.
IPAQ-SF	9837	1401.3	1817.6	0	56,826
<600 MET (Low activity) (n/%)	3906/39.7				
600–3000 MET (n/%) (Moderate activity)	4770/48.5				
>3000 MET (n/%) (High activity)	1161/11.8				
FRS	9836	−0.49	1.51	−7.0	8.0
FFaMES Total Score	9838	94.8	10.3	32	144
Non-reactivity Score	9838	34.2	8.5	9.0	64.0
Non-judging Score	9838	28.8	7.1	8.0	55.0
Awareness of internal cues Score	9838	14.3	5.1	6.0	35.0
Awareness of external cues Score	9838	16.7	5.2	6.0	35.0

IPAQ-SF: International Physical Activity Questionnaire—Short Form; MET: Metabolic Equivalent of Task (minutes/week); FFaMES: The Four Facet Mindful Eating Scale; SD: Standard Deviation; Avg: average; Min.: Minimum; Max.: Maximum.

**Table 3:** Spearman Correlation Coefficients among Physical Activity (IPAQ MET), Body Dissatisfaction (FRS), and Mindful Eating (FFaMES) Variables.

	1. IPAQ-SF (MET)	2. FRS	3. FFaMES Total Score	4. FFaMES Non-Reactivity Score	5. FFaMES Non-Judging Score	6. FFaMES Internal Cues Score	7. FFaMES External Cues Score
1. IPAQ (MET)	—						
2. FRS	0.065 **	—					
3. FFaMES Total Score	0.066 **	−0.013	—				
4. FFaMES Non-reactivity Score	0.031 **	0.001	0.679 **	—			
5. FFaMES Non-judging Score	0.010	0.027 **	0.663 **	0.671 **	—		
6. FFaMES Awareness of Internal cues Score	0.009	0.014	−0.256 **	−0.619 **	−0.592 **	—	
7. FFaMES Awareness of external cues Score	0.037 **	−0.101 **	0.037 **	−0.424 **	−0.391 **	0.505 **	—

Note. Values represent Spearman’s rho (ρ) correlation coefficients. ** Correlation is significant at the 0.01 level (2-tailed). IPAQ-SF: International Physical Activity Questionnaire—Short Form; MET: Metabolic Equivalent of Task (minutes/week); FFaMES: The Four Facet Mindful Eating Scale.

**Table 4:** Differences in Body Dissatisfaction (FRS) and Mindful Eating (FFaMES) Scores across Physical Activity Categories Based on Kruskal–Wallis Tests.

Variable	Low PA (Mean ± SD)	Moderate PA (Mean ± SD)	High PA (Mean ± SD)	χ^2^	*p*	Pairwise Comparisons
FRS	−0.58 ± 1.62	−0.49 ± 1.44	−0.27 ± 1.38	χ^2^(2) = 46.64	<0.001	Low-Moderate (*p* = 0.005); Low-High (*p* < 0.001); Moderate-High (*p* < 0.001)
FFaMES Total Score	94.30 ± 10.17	95.27 ± 10.38	94.91 ± 10.93	χ^2^(2) = 43.46	<0.001	Low-Moderate (*p* < 0.001); others ns
FFaMES Non-reactivity Score	34.00 ± 8.63	34.53 ± 8.36	34.02 ± 8.73	χ^2^(2) = 14.37	<0.001	Low-Moderate (*p* < 0.001); others ns
FFaMES Non-judging Score	28.93 ± 7.18	28.92 ± 7.12	28.45 ± 7.18	χ^2^(2) = 1.47	0.480	No significant differences
FFaMES Internal cues Score	14.18 ± 5.10	14.32 ± 5.09	14.80 ± 5.50	χ^2^(2) = 0.31	0.857	No significant differences
FFaMES External cues Score	16.45 ± 5.31	17.07 ± 5.10	16.64 ± 5.61	χ^2^(2) = 11.01	0.004	Low-Moderate (*p* = 0.011); others ns

Mean: Average; SD: Standard Deviation; ns = not significant. All *p*-values are Bonferroni-adjusted. FFaMES: The Four Facet Mindful Eating Scale, FRS: Figure Rating Scale.

**Table 5:** Hierarchical Regression Analysis Predicting Physical Activity (IPAQ MET) from Demographic, Socioeconomic, and Psychosocial Variables.

	β	t	*p*
Model 1 (Controls)			
Age	0.01	1.12	0.259
Gender (1 = Female)	−0.10	−9.91	<0.001
BMI	−0.02	−1.35	0.176
Education (ref = others)			
Primary	0.01	0.13	0.895
High school	0.01	1.29	0.195
Master	−0.00	−0.17	0.864
Income status	−0.02	−1.97	0.049 *
Model 2 (Controls + Psychosocial)			
FRS	0.02	2.30	0.021 *
FFaMES Total Score	0.06	2.93	0.003 **

Note: R^2^ Model 1 = 0.033, R^2^ Model 2 = 0.035, ΔR^2^ = 0.002, *p* = 0.005, * *p* < 0.05, ** *p* < 0.01.

## Data Availability

The datasets used and/or analysed during the current study are available from the corresponding author on reasonable request.

## Supplementary Materials

The following supporting information can be downloaded at https://www.mdpi.com/article/10.3390/nu18081292/s1: Table S1: Spearman correlation coefficients among physical activity (IPAQ-MET), body dissatisfaction (FRS), and mindful eating (FFaMES total score) in women; Table S2: Spearman correlation coefficients among physical activity (IPAQ-MET), body dissatisfaction (FRS), and mindful eating (FFaMES total score) in men; Table S3: Hierarchial regression analysis predicting physical activity (IPAQ-MET) in women; Table S4: Hierarchial regression analysis predicting physical activity (IPAQ-MET) in men.

## Abbreviations

Avg	Average
BMI	Body mass index
CBS	Current Body size
FFaMES	The Four Facet Mindful Eating Scale
FRS	Figure Rating Scale
IBS	Ideal body size
IPAQ-LF	The International Physical Activity Questionnaire-Long Form
IPAQ-SF	The International Physical Activity Questionnaire-Short Form
Max	Maximum
METs	Metabolic Equivalent of Task
Min	Minimum
NCD	Noncommunicable disease
SD	Standard deviation
