# Supplementary material for: Associations Between Physical Activity, Body Dissatisfaction, and Mindful Eating in Turkish Adults
Source: Nutrients. 2026 Apr 20;18(8):1292. doi: 10.3390/nu18081292 (PMC13118447; doi:10.3390/nu18081292)
Supplement: Supplementary file 1 [file nutrients-18-01292-s001.zip › nutrients-4181053-supplementary.pdf]

Supplementary Table S1. Spearman correlation coefficients among physical activity (IPAQ-MET), body dissatisfaction (FRS), and mindful eating (FFaMES total score) in women

	**1. IPAQ (MET)**	**2. FRS**	**3. FFaMES Total Score**
**1. IPAQ (MET)**	—		
**2.FRS**	.065**	—	
**3. FFaMES Total Score**	.042**	.000	—

Note: Values represent Spearman's rho coefficients. $p < .05$, ** $p < .01$. IPAQ-MET: International Physical Activity Questionnaire (MET-min/week); FRS: Figure Rating Scale; FFaMES: Four-Facet Mindful Eating Scale.

Supplementary Table S2. Spearman correlation coefficients among physical activity (IPAQ-MET), body dissatisfaction (FRS), and mindful eating (FFaMES total score) in men

	**1. IPAQ (MET)**	**2. FRS**	**3. FFaMES Total Score**
**1. IPAQ (MET)**	—		
**2.FRS**	.065**	—	
**3. FFaMES Total Score**	.066**	-.037*	—

Note: Values represent Spearman's rho coefficients. $p < .05$, ** $p < .01$. IPAQ-MET: International Physical Activity Questionnaire (MET-min/week); FRS: Figure Rating Scale; FFaMES: Four-Facet Mindful Eating Scale.

Supplementary Table S3. Hierarchical regression analysis predicting physical activity (IPAQ-MET) in women

	**β**	**t**	**P**
**BMI**	-0.017	-1.10	.273
**Education**	–.0.002	–0.09	.925
**Income status**	-0.018	-1.34	.182

**Body dissatisfaction (FRS)**	0.015	1.13	.258
**Mindful eating (FFaMES total score)**	0.006	0.44	.662

Note: $R^2 = .012$, $p < .001$ BMI: Body Mass Index, FRS: Figure Rating Scale; FFaMES: Four-Facet Mindful Eating Scale.

Supplementary Table S4. Hierarchical regression analysis predicting physical activity (IPAQ-MET) in men

	**β**	**t**	**P**
**BMI**	-0.008	-0.46	.648
**Education**	0.016	0.85	.394
**Income status**	0.027	1.68	.094
**Body dissatisfaction (FRS)**	0.029	1.73	.083
**Mindful eating (FFaMES total score)**	0.046	2.95	.003

Note: $R^2 = .060$, $p < .001$ BMI: Body Mass Index FRS: Figure Rating Scale; FFaMES: Four-Facet Mindful Eating Scale.
